# Editorial

**Published:** 1875-01

**Authors:** 


### Editorial.

### TWENTY-NINTH VOLUME.

With this number begins the twenty-ninth volume of the DENTAL REGISTER; and the nineteenth year of the present Editor's connection with, and work upon it.

This work, through all these years, though laborious and incessant, has been in many respects a pleasant one, and not without a good measure of gratification.

Through all the vicissitues and changes that have occurred during that time, it has never missed the regular issue of a single number, though for several years it was carried on at a considerable, pecuniary sacrifice, but a compensation for this, to some extent at least, was realized in the hope that some good was being accomplished. The REGISTER has always had many true friends, and some who thought it was no better than it ought to be. We have had our full share of the ups and downs of an editor's life; all of which we have endeavored to accept, with a becoming demeanor. We trust that for the future the REGISTER may present some improvement upon the past, and this will result if the profession desire to have it so.

### OBITUARY.

### DEATH OF DR. GEORGE E. HAWES, OF NEW YORK.

A meeting of dentists, called at a few hours' notice, on Tuesday afternoon, August 25th, 1874, (on receipt of the news of the death of Dr. George E. Hawes, of this city, at Wrentham, Mass.,) was held at the residence of Dr. J. G. Ambler, No. 25 West Twenty-third street, who presented the following preamble and resolutions, which were unanimously adopted:

WHEREAS, An All-wise Providence has seen fit to remove, by death, our much esteemed friend and professional brother, Dr. George E. Hawes, of this city; therefore,

*Resolved*, That while we bow in humble submission to the decrees of Him who doeth all things well, we can not refrain from expressing our feelings of sadness and sorrow at the loss our profession has sustained in the demise of our much esteemed friend and brother.

*Resolved*, That we show our affection and appreciation for his many virtues and bright example, by placing on record these expressions of our bereavement and sorrow.

*Resolved*, That our sympathies, true and heartfelt, are hereby tendered to the family and friends of deceased, as well as the profession at large, in this dispensation of Providence. Though we lament his death, we can not be unconscious that our loss is his gain.

*Resolved*, That a committee be appointed by the chair to prepare a suitable memorial for publication in the dental and medical journals.

*Resolved*, That these proceedings be published in the New York Times and the Observer; also, a copy sent to the family of deceased. [Signed,]

J. G. AMBLER, Secretary. JOHN ALLEN, Chairman.

*Committee on Memorial.*

J. G. AMBLER, JOHN ALLEN, W. H. ATKINSON, J. PARMLEY, L. COVILLE.

### MEMORIAL
### OF
### DR. GEORGE E. HAWES.

Dr. George E. Hawes, the subject of this brief memorial, was born in Wrentham, Massachusetts, May 28, 1810. His father, Colonel George Hawes, was a man of integrity and uprightness, whose patriotism was of an active character; in our Revolutionary struggles, he filling his place in the ranks of the colonial patriots, battling against aggressive and oppressive acts of the mother country, and subsequently serving the State in its halls of Legislation. His mother still survives him at the advanced age of ninety-five years, having laid all the members of her immediate family in the resting-place of the dead. Dr. Hawes commenced his preparatory course as a dentist in the office of the late Dr. John Lovejoy, and after completing his course of study with him, he commenced the practice of his profession in Park Place, and after years of diligent and successful practice, located in Bond street, where he continued his professional career until laid aside by impaired health. He was an eminently successful dentist, retaining the confidence of his patients by the perfection of his operations and his uniform urbanity.

By the members of the profession he was universally esteemed; his opinions on different operations, or cases in practice, were appreciated, because they were judicious and sound and there are none who knew him who did not feel that a safe operator has been lost by the community, and an appreciative and safe counsellor by the profession. We shall miss his acquaintence and counsel. Dr. Hawes was a Christian gentleman who adorned the circle in which he moved, and those who knew him best, appreciated him most.

The family who survive him have, in the recolection of the past, and in the assurances of his blessed future, all the consolation necessary to assuage their great grief and make supportable their great bereavement.

[Signed,] J. G. AMBLER, *On behalf of the Committee.*

---

DIED on the 14th of Dec., in the 78th year of his age Dr. E. Parmly of New York.

Dr. Parmly has been an active and prominent member of the dental profession for about fifty years; and in the earliest part of his professional life he did more perhaps than any other man, in this country at least, to give tone, dignity and identity to the Dental Profession. He was one of nature's true noblemen, to which was added a high degree of refined culture. We purpose as early as practicable, perhaps in the next No. of the REGISTER, to publish a sketch of his life.

